# The Potential of Congo Red Supplied Aggregates of Multitargeted Tyrosine Kinase Inhibitor (Sorafenib, BAY-43-9006) in Enhancing Therapeutic Impact on Bladder Cancer

**DOI:** 10.3390/ijms25010269

**Published:** 2023-12-23

**Authors:** Małgorzata Lasota, Daniel Jankowski, Anna Wiśniewska, Michał Sarna, Marta Kaczor-Kamińska, Anna Misterka, Mateusz Szczepaniak, Joanna Dulińska-Litewka, Andrzej Górecki

**Affiliations:** 1 Chair of Medical Biochemistry, Jagiellonian University Medical College, Kopernika 7, 31-034 Krakow, Poland; marta.b.kaczor@uj.edu.pl (M.K.-K.); anna.misterka@uj.edu.pl (A.M.); joanna.dulinska-litewka@uj.edu.pl (J.D.-L.); 2 SSG of Targeted Therapy and Supramolecular Systems, Jagiellonian University Medical College, Kopernika 7, 31-034 Krakow, Poland; daniel.jankowski@student.uj.edu.pl (D.J.); mateusz.szczepaniak@student.uj.edu.pl (M.S.); 3 Department of Physical Biochemistry, Faculty of Biochemistry, Biophysics and Biotechnology, Jagiellonian University, Gronostajowa 7, 30-387 Krakow, Poland; andrzej.gorecki@uj.edu.pl; 4 Chair of Pharmacology, Faculty of Medicine, Jagiellonian University Medical College, Grzegórzecka 16, 31-531 Krakow, Poland; anna.niepsuj@uj.edu.pl; 5 Department of Biophysics, Faculty of Biochemistry, Biophysics and Biotechnology, Jagiellonian University, Gronostajowa 7, 30-387 Krakow, Poland; michal.sarna@uj.edu.pl

**Keywords:** bladder cancer, tyrosine kinases inhibitor, Congo red, personalized medicine, multidisciplinary treatment

## Abstract

Bladder cancer is a common malignancy associated with high recurrence rates and potential progression to invasive forms. Sorafenib, a multi-targeted tyrosine kinase inhibitor, has shown promise in anti-cancer therapy, but its cytotoxicity to normal cells and aggregation in solution limits its clinical application. To address these challenges, we investigated the formation of supramolecular aggregates of sorafenib with Congo red (CR), a bis-azo dye known for its supramolecular interaction. We analyzed different mole ratios of CR-sorafenib aggregates and evaluated their effects on bladder cancer cells of varying levels of malignancy. In addition, we also evaluated the effect of the test compounds on normal uroepithelial cells. Our results demonstrated that sorafenib inhibits the proliferation of bladder cancer cells and induces apoptosis in a dose-dependent manner. However, high concentrations of sorafenib also showed cytotoxicity to normal uroepithelial cells. In contrast, the CR-BAY aggregates exhibited reduced cytotoxicity to normal cells while maintaining anti-cancer activity. The aggregates inhibited cancer cell migration and invasion, suggesting their potential for metastasis prevention. Dynamic light scattering and UV-VIS measurements confirmed the formation of stable co-aggregates with distinctive spectral properties. These CR-sorafenib aggregates may provide a promising approach to targeted therapy with reduced cytotoxicity and improved stability for drug delivery in bladder cancer treatment. This work shows that the drug-excipient aggregates proposed and described so far, as Congo red—sorafenib, can be a real step forward in anti-cancer therapies.

## 1. Introduction

Bladder cancer is the second most frequently diagnosed cancer of the genitourinary system in Europe, characterized by frequent recurrences and a high risk of progression [1,2,3,4]. This tumor most often originates from the epithelium of the transitional layer covering the urinary tract, also known as the urothelial epithelium, and is responsible for about 90–95% of urinary tract cancers [5,6]. Bladder cancer diagnosed in most patients is in a noninvasive form; however, the recurrence rate after treatment with transurethral electro resection reaches up to 50–70% after 5 years [7]. The reasons for the high rate of bladder cancer are unknown. Importantly, in about 40% of patients who were initially diagnosed with noninvasive bladder cancer—the disease progresses to invasive cancer [8,9]. One of the symptoms of bladder cancer is hematuria, which is common in advanced forms of the disease [5,10,11]. Prolonged time from the moment of first symptoms occurrence to the diagnosis of bladder cancer probably negatively influences the treatment results. Research shows that increased activation of receptor tyrosine kinases (RTK), including the platelet-derived growth factor receptor (PDGFR), c-KIT receptor, epidermal growth factor receptor (EGFR), or vascular endothelial growth factor receptor (VEGFR) [12,13,14,15,16] and the intracellular PI3K/AKT/mTOR pathway [17,18,19] may play a huge role in the development of bladder cancer. For these reasons, multitargeted tyrosine kinase inhibitors (e.g., sorafenib, Nexavar, BAY-43-9006, BAY) are used as compounds in anti-cancer therapy.

Sorafenib is used to treat advanced renal cell carcinoma [20,21], hepatocellular carcinoma [22,23] that cannot be treated with surgery, and differentiated thyroid cancer [24,25]. Sorafenib directly blocks the autophosphorylation of many receptor tyrosine kinases, such as VEGFR1, 2 and 3, PDGFRβ, c-Kit, and RET, and inhibits the serine-threonine kinase Raf. For these reasons, the broad spectrum of action of this inhibitor may ultimately block RTKs involved not only in tumorigenesis (Flt-3, c-Kit, RET) but also in angiogenesis (VEGFR1, 2, 3 and PDGFRβ) [26]. Previous studies show that low concentrations (<1 µM) of this compound may promote proliferation and migration of bladder cancer cells. Only a higher concentration (≥3 µM) of sorafenib revealed its inhibitory effect on ERK1/2 phosphorylation, migration, and proliferation of cancer cells [27]. Its inhibitory effect on the growth and survival of bladder cancer cells has also been confirmed by other studies. Unfortunately, such high doses (IC50 of about 11 µM) [28] may prove to be cytotoxic to normal cells. The results of our studies have shown that sorafenib at a concentration above 10 µM causes a cytotoxic effect on normal cells in the SV-HUC-1 line.

In addition, sorafenib itself forms large aggregates (drug-based colloids) within minutes to hours after its formation, limiting its potential use for drug delivery [29,30,31,32]. For this reason, the technique of drug administration is extremely important in its target transport. One of the most interesting and innovative compounds that can facilitate drug delivery are supramolecular structures formed as a result of self-association [33]. An example of such a structure is Congo red (CR). This compound is known as a dye, e.g., in the diagnosis of amyloidosis. However, its supramolecular structure and the ability to react with anti-cancer compounds is undoubtedly an innovative approach in targeted therapy [34,35]. Studies show that well-known colloidal drug aggregators (including sorafenib) can be co-formulated with bis-azo dyes to form colloids that are much more stable and much more monodisperse than colloidal aggregates. Very importantly, the new co-formulated colloids can still adsorb protein, inhibit a number of enzymes, and, indeed, remain stable in high-protein environments similar to those used in typical cell cultures [29,31].

For these reasons, the aim of our work was to analyze several mole ratios of the supramolecular assembling of the bis-azo dye (Congo red) with the aggregating anti-cancer drug (sorafenib) and to evaluate the comparison of the drug alone and its combination with Congo red (co-aggregates) on selected properties of bladder cancer cells with different malignant potential. Furthermore, we also evaluated the effect of the test compounds on normal cells of the SV-HUC-1 line.

## 2. Results

### 2.1. Effect of Sorafenib (Alone) and in Aggregates with Congo Red on the Viability and Survival of Bladder Cancer Cells

We examined the effect of sorafenib, a multikinase tyrosine kinase inhibitor, both alone and in aggregates with Congo red, on various grades of bladder cancer cell lines (RT4, T24). Sorafenib was added to the culture medium at concentrations from 0.5 μM to 75 μM.

**Figure 1:**
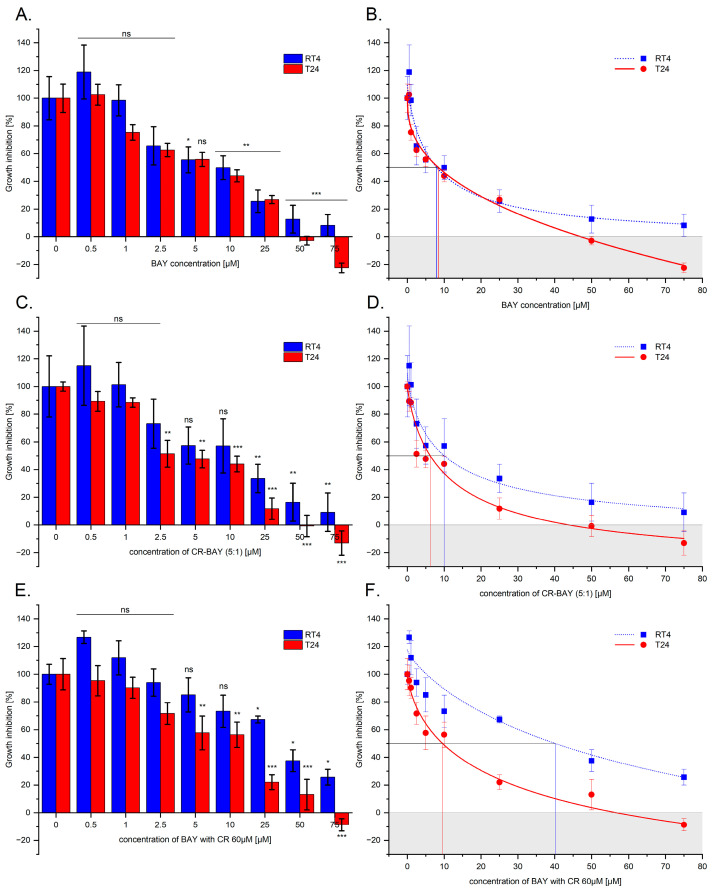
Effect of sorafenib alone and in aggregates with Congo red on bladder cells (RT4 and T24). The dose-dependent effect of sorafenib alone and in aggregates with Congo red on the viability of RH30 and RD cells after 48 h incubation (**A**,**C**,**E**). Statistical significance between non-treated and treated samples was evaluated using ANOVA with Dunnett post-test: ns—non-significant (*p* > 0.05) in comparison with a control sample (without investigated compounds); * 0.01 < *p* < 0.05, ** 0.001 < *p* < 0.01, ***—*p* < 0.001 Growth inhibition curve in standard culture conditions (**B**,**D**,**F**).

The exposure of RT4 and T24 cells to sorafenib resulted in significant dose-dependent suppression of viability, evaluated with MTS assay, compared to the control cultures (i.e., non-treated cells), as shown in Figure 1A and Supplementary Figures S1 and S2. At the highest concentrations of sorafenib, complete inhibition of growth was observed in T24 cells after 48 h of incubation with the test inhibitor. The absorbance value was below the value obtained for day 0, which may indicate a cytotoxic effect of the test compound. In the case of RT4 cells, for a concentration of 75 µM, the cell growth fell below 10% of control growth, and the difference from day 0 was not statistically significant. As a result of extending the incubation time to 72 h, an increase in the observed cytostatic effect was observed (Supplementary Figure S2).

A 50% and 90% inhibition of the cell growth was determined by fitting a sigmoidal model of the dose-dependent curve obtained for sorafenib (Figure 1B and Table 1).

Next, we tested the use of sorafenib in an aggregate with Congo red at an optimal molar ratio of 5:1 (Figure 1C) and at a constant CR concentration of 60 µM (Figure 1E). In the case of CR-BAY 5:1 aggregate, we observed a similar cytostatic effect as for the inhibitor alone (Figure 1A). The obtained IC50 and IC90 values for the CR-BAY 5:1 aggregates were lower than for the inhibitor alone (the exception was the IC50 value for the RT4 line). However, at a constant Congo red concentration of 60 µM and the tested range of inhibitor concentrations, we observed a decrease in the observed cytostatic effect and a significant increase in the determined IC50 and IC90 values.

**Figure 2:**
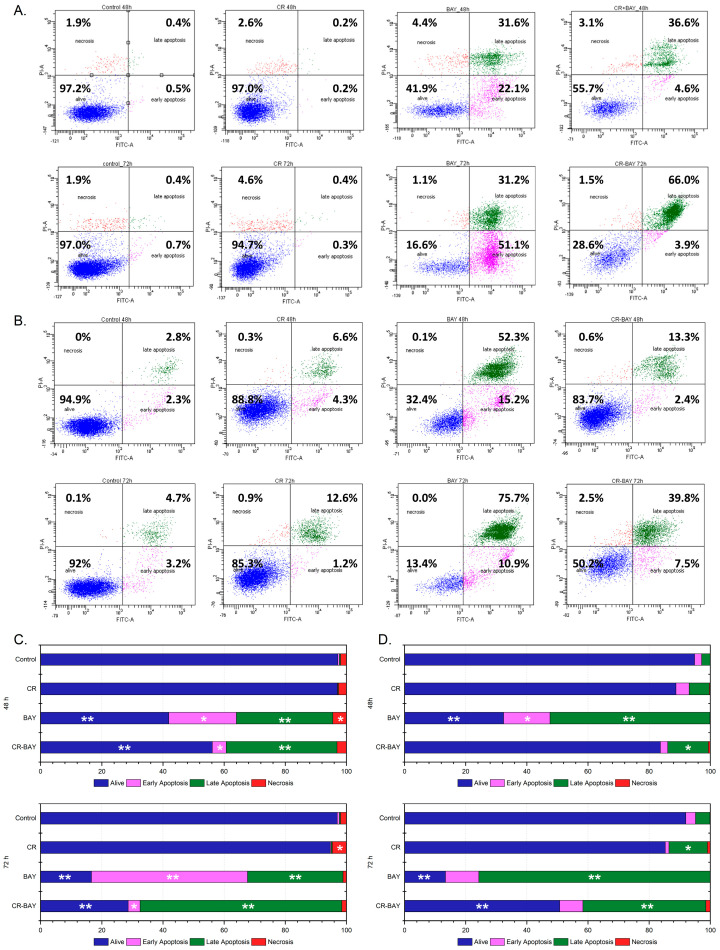
The effect of sorafenib on apoptosis and necrosis of RT4 and T24 cells. The images show flow cytometry analysis of Annexin-V and PI staining presented on a dot-plot graph. Graphic representation of four cell states: alive—the lower left square; cells undergoing necrosis—the upper left square; cells in early apoptosis—the right lower square; and cells in late apoptosis the upper right square. RT4 cells were incubated with BAY (at the IC50 concentration) and CR-BAY 5:1 (**A**). T24 cells were incubated with BAY (at the IC50) and CR-BAY 5:1 (**B**). Cumulative bar charts show the inter-relation between the state of RT4 cells after 48 h and 72 h exposure to BAY and CR-BAY 5:1 (**C**). Cumulative bar charts show the inter-relation between the state of T24 cells after 48 h and 72 h exposure to BAY and CR-BAY 5:1 (**D**). Statistical significance between investigated samples was evaluated using a Mann–Whitney test (only statistically significant data were marked): * 0.01 < *p* < 0.05, ** 0.001 < *p* < 0.01).

For these reasons, we used an aggregate of sorafenib with Congo red in a 5:1 ratio for further studies. We tested the effect of the inhibitor itself and the tested aggregate on the survival of the bladder cancer cell lines using Annexin V and propidium iodide (Figure 2).

The results of our study showed a significant increase in the percentage of apoptotic cells compared to controls in both tested cell lines treated with sorafenib (Figure 2). After extending incubation with the inhibitor to 72 h, a further decrease in tumor cell survival and an increase in apoptotic cells were observed. 

Also, treatment of cells with sorafenib aggregate with Congo red (ratio 5:1) resulted in an increase in the percentage of apoptotic cells relative to the control for both tested cell lines, although the observed effect was less than with the drug alone. Interestingly, the supramolecular dye alone (Congo red) did not cause apoptosis of tumor cells, regardless of the incubation time.

Additionally, we presented the dependence of sorafenib- and CR-BAY-induced apoptosis on caspase 3 in Supplementary Figure S3.

### 2.2. Effect of Sorafenib (Alone) and in Aggregate with Congo Red (5:1) on Human Uroepithelial SV-HUC-1

**Figure 3:**
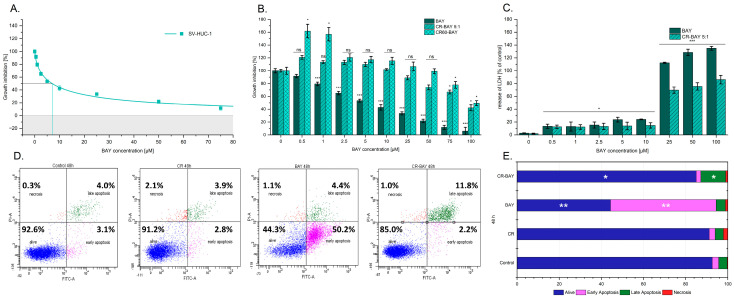
Effect of sorafenib alone and in aggregates with Congo red on human uroepithelial cells (SV-HUC-1). Growth inhibition curve (**A**). The dose-dependent effect of sorafenib alone and in aggregates with Congo red (CR60-BAY, CR-BAY 5:1) on the viability of SV-HUC-1 cells after 24 h incubation, Gray shading indicates negative values. The lines indicate the IC50 value (**B**). The cytotoxic influence of BAY-43-9006 and in aggregate with Congo red (5:1) by LDH test (results presented as % LDH released relative to control). Statistical significance between non-treated and treated samples was evaluated using ANNOVA with Dunnett post-test: ns—non-significant (*p* > 0.05) in comparison with a control sample (without BAY or CR-BAY); * 0.01 < *p* < 0.05, ** 0.001 < *p* < 0.01, *** *p* < 0.001 (**C**). The effect of BAY-43-9006 and in aggregate with Congo red (5:1) on apoptosis and necrosis of human uroepithelial cells (**D**,**E**).

We also tested the effect of the investigated compounds on normal human uroepithelial cells (line SV-HUC-1). The results obtained showed that sorafenib caused a significant dose-dependent suppression of viability as assessed by the MTS assay compared to the control cultures (Figure 3A,B). The cytostatic half-dose value was 7.07 ± 0.08 µM. Interestingly, in the case of the tested CR60-BAY and CR-BAY (5:1) aggregates, we observed growth inhibition only at high concentrations of the tested inhibitor above 50 µM.

Moreover, after 24-h incubation, it was observed that BAY-43-9006 at concentrations of 0.5–10 μM showed no cytotoxicity to normal uroepithelial cells Figure 3C. An increased LDH efflux was observed at concentrations of 50 µM, 75 µM, and 100 µM. These results showed that tested compounds have low toxicity to human uroepithelial cells. 

The cytometry test used, which involved Annexin V-propidium iodide staining, showed that sorafenib causes a significant increase in the percentage of early apoptosis cells compared to untreated cells. Interestingly, our studies have shown that the tested CR-BAY aggregate (5:1) has little effect on the survival of normal cells, causing only a slight increase in apoptotic cells. The observed effect is clearly smaller than that observed for the inhibitor alone (Figure 3D,E).

Furthermore, as in the case of bladder cancer cells, no significant effect of the carrier itself on their survival was found for normal SV-HUC-1 cells.

### 2.3. BAY-43-9006 Inhibitor and Its Congo Red Aggregates Diminishes Migration and Invasion of Bladder Cancer Cells

**Figure 4:**
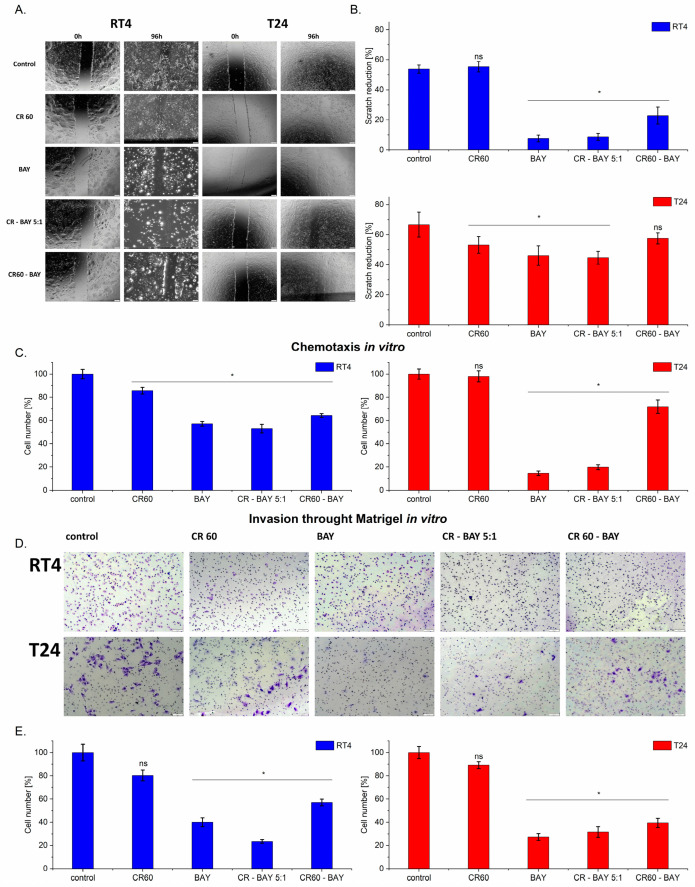
Effect of sorafenib alone and in aggregates with Congo red on the motility of bladder cancer cell lines. Imaging under a 4× objective (**A**) Exposure to sorafenib and aggregates CR-BAY inhibited migration of bladder cancer cell lines in a scratch assay observed after 96 h. (**B**) Percentage of scratch reduction after 96 h (n = 3). (**C**) In a chemotactic assay, cells incubated with BAY and CR-BAY showed reduced migration towards 10% FBS, n = 3. (**D**) Representative images of bladder tumor cell invasion by Matrigel to 10% FBS in vitro show reduced cell invasion capacity under the influence of sorafenib and aggregates CR-BAY. Imaging under a 10× objective (**E**) Percentage of invasive cells after passing through Matrigel compared to the control, n = 4, Statistical analysis: ns—non-significant (*p* > 0.05), * *p* < 0.05. Data in graphs are represented as mean +/− SD.

Bladder cancer cells were treated with Congo red, BAY-43-9006 inhibitor, and aggregates (CR60-BAY, CR-BAY 5:1), and cell migration was assessed by the wound healing test/scratch test (Figure 4A,B). Optical images show scratch overgrowth with time for bladder cancer cells (Figure 4A). The photographs were captured immediately after wounding as well as 96 h later with the corresponding control (non-treated cells). A significant reduction in cell motility was observed after exposure to both BAY and CR-BAY 5:1 in the tested cell lines (Figure 4A,B).

Non-treated cells and cells incubated with Congo red (alone) almost entirely closed the wound in approximately 96 h, while the scratch remained open in cells treated with BAY and CR-BAY 5:1, regardless of the cell type. These results indicate that the motility of the RD and T24 cell lines was significantly inhibited by the presence of BAY and CR-BAY 5: 1 after 96 h. The scratch reduction effect was similar for both cell lines tested, except for the incubation of cells with CR60-BAY. Only in the case of the RT4 line was a significant decrease in migration observed under the influence of this aggregate.

Additionally, chemotaxis of bladder cancer cells towards FBS gradient through 8 μm pores was evaluated. Treatment of cells with BAY and CR-BAY 5:1 24 h before the experiment significantly inhibited chemotaxis of tumor cells towards FBS (Figure 4C), indicating their impaired migratory capabilities.

Next, we evaluated the invasion of bladder tumor cells by Matrigel to 10% FBS in vitro (4DE. Before the experiment, RT4 and T24 cells were treated with sorafenib at IC50 concentration or CR-BAY aggregates for 24 h. We observed reduced cell invasion capacity under the influence of sorafenib and the tested CR-BAY aggregates, regardless of the cell lines.

### 2.4. Effect of Sorafenib (Alone) and in Aggregate with Congo Red on the Cytoskeleton and Nanomechanical Properties of Bladder Cancer Cells

**Figure 5:**
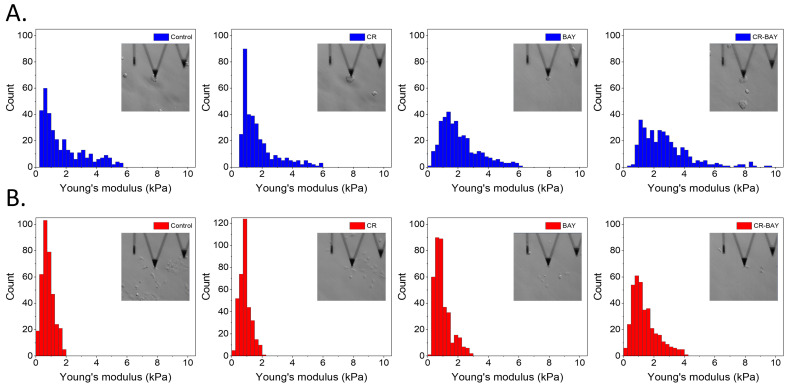
Histograms of the Young’s modulus values for RT4 (**A**) and T24 (**B**) cells. Inset images show cells during nanomechanical analysis.

**Figure 6:**
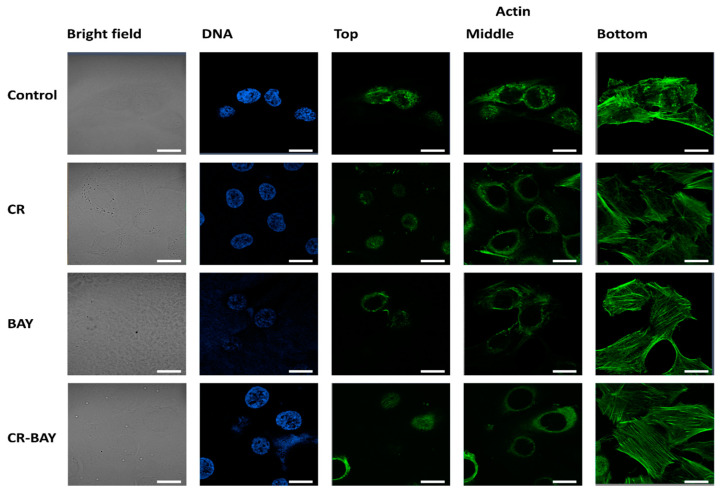
Representative confocal microscopy images of the RT4 cells. Scale bar of all images represent 20 µm.

**Figure 7:**
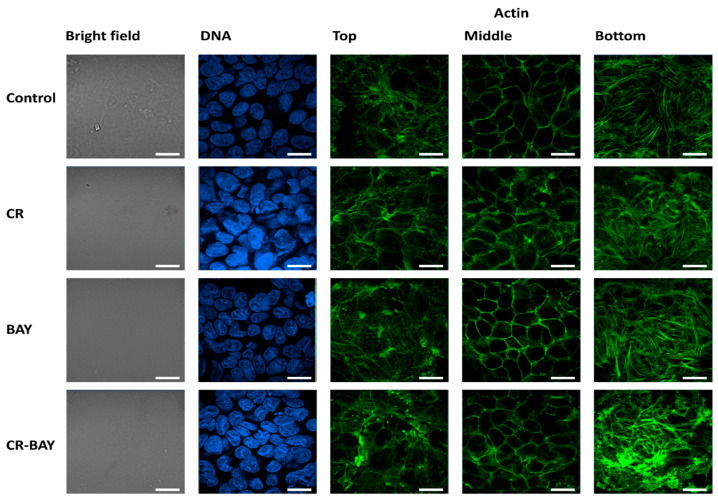
Representative confocal microscopy images of the T24 cells. Scale bar of all images represent 20 µm.

Nanomechanical analysis of cells indicated that control RT4 cells had an average value of the Young’s modulus (E) of 1.78 ± 1.39 kPa, whereas T24 cells had an average E value of 0.81 ± 1.38 kPa (Figure 5). As expected, more aggressive cells (T24) had significantly smaller E values than less aggressive cells (RT4). Confocal microscopy analysis of the actin cytoskeleton of the cells revealed that both RT4 and T24 cells had short actin fibers at the bottom of the cells, with cortical actin present in the middle and top of the cells (Figure 6 and Figure 7). It should be noticed that RT4 cells had significantly more actin in the cell cortex when compared to T24 cells, which would explain the higher values of the elastic modulus for the cells. Congo red had no significant effect on the cell elasticity and organization of the actin cytoskeleton in both RT4 and T24 cells. RT4 cells treated with Congo red had an average E value of 1.75 ± 1.17 kPa, whereas T24 cells treated with Congo red had an average E value of 0.88 ± 0.39 kPa. On the other hand, RT4 cells treated with sorafenib alone had an average E value of 2.11 ± 1.26 kPa, whereas T24 cells treated with sorafenib alone had an average E value of 0.97 ± 0.55 kPa. Both cells started to develop stress fibers at the bottom of the cells. Finally, RT4 cells treated with sorafenib in aggregate with Congo red had an average E value of 2.76 ± 1.65 kPa, whereas T24 cells treated with sorafenib in aggregate with Congo red had an average E value of 1.41 ± 0.84 kPa. Both cell types had developed distinct stress fibers following treatment. Interestingly, the response of T24 cells to sorafenib in aggregate with Congo red was greater than for RT4 cells. For RT4 cells, the increase in the elastic modulus was approximately 155% compared to control cells, while for T24 cells, there was a 174% increase.

### 2.5. Sorafenib and Its Congo Red Aggregates Affect the Phosphorylation Level of Akt and Erk1/2 Kinase in Bladder Cancer Cells

Subsequently, the effect of the BAY-43-9006 inhibitor and aggregates with CR on downstream mediators was evaluated.

**Figure 8:**
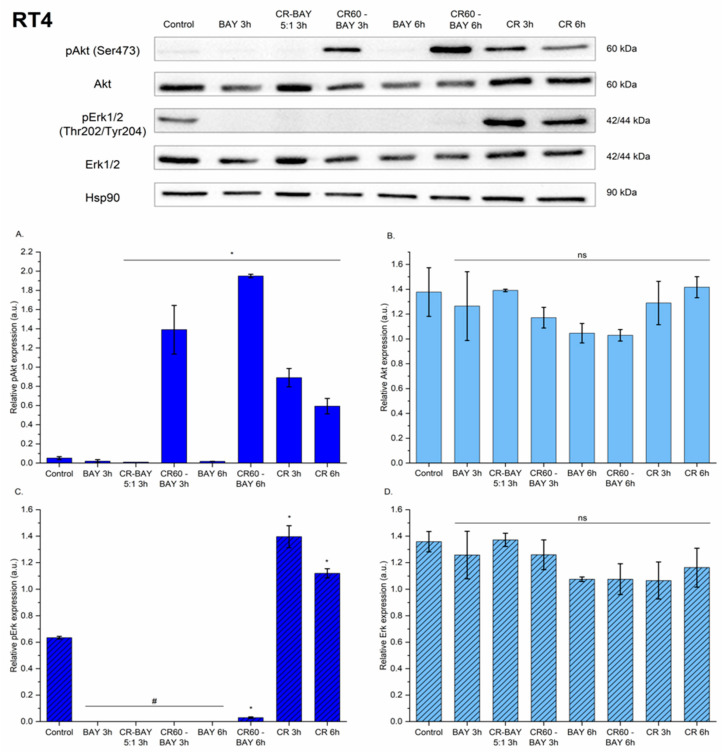
The effect of investigated compounds on the p-AKT, AKT, p-ERK, and ERK protein levels in the RT4 cell line after 3 and 6 h of incubation with inhibitors (at the IC50 concentration). Results show representative Western blot images with immunodetection and densitometric evaluation of the bands (**A**–**D**), #-expression of protein was not detected. Statistical significance between non-treated and treated samples was evaluated using ANOVA with Dunnett post-test: ns—non-significant (*p* > 0.05), * 0.01 < *p* < 0.05.

Treatment of RT4 cells with sorafenib or CR-BAY 5:1 decreased Akt phosphorylation at Ser473 (Figure 8A). However, incubation of RT4 cells with Congo red alone or CR60-BAY resulted in an increase in pAkt phosphorylation. The expression of pErk1/2 (Thr202/Tyr204) was not detected in RT4 cell lines incubated with sorafenib, CR-BAY (5:1) and CR60-BAY (3 h). When incubation of CR60-BAY cells was extended to 6 h, a decrease in pERK1/2 phosphorylation was observed compared to the control (untreated cells). There was no significant difference between treated and control RT4 cells in total Akt and Erk1/2 (Figure 8B,D).

**Figure 9:**
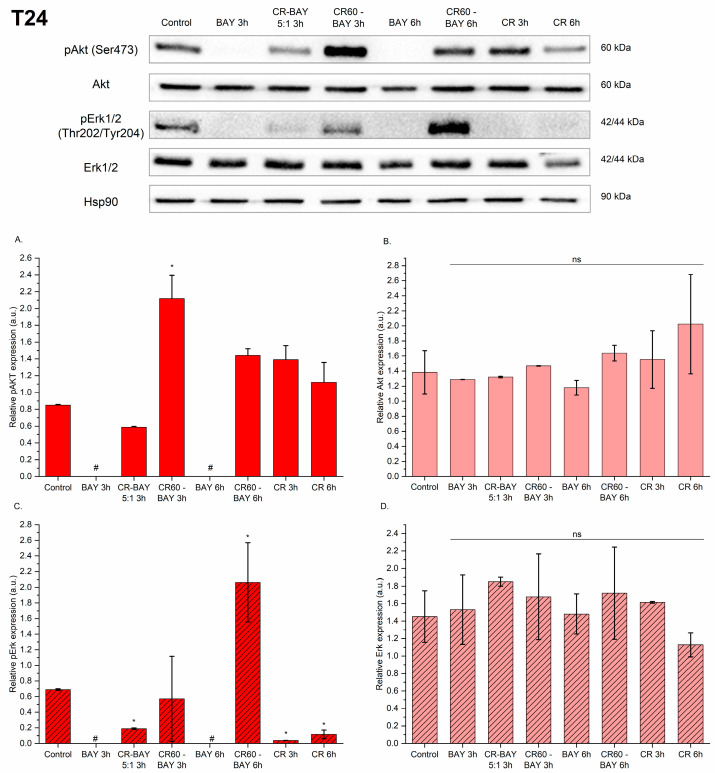
The effect of investigated compounds on the p-AKT, AKT, p-ERK, and ERK protein level in the T24 cell line after 3 and 6 h of incubation with inhibitors (at the IC50 concentration). Results show representative Western blot images with immunodetection and densitometric evaluation of the bands (**A**–**D**), #—expression of protein was not detected. Statistical significance between non-treated and treated samples was evaluated using ANOVA with Dunnett post-test: ns—non-significant (*p* > 0.05), * 0.01 < *p* < 0.05.

Expression of pAkt and pErk1/2 was not detected in T24 cells treated for 3 or 6 h with sorafenib (Figure 9A,C). Treatment T24 with CR-BAY 5:1 decreased Akt phosphorylation at Ser473. As in the cells of the RT4 line, also in the case of T24, we observed an increase in Akt phosphorylation under the influence of Congo red alone or CR60-BAY. Interestingly, we observed a decrease in Erk1/2 phosphorylation in cells treated with CR-BAY (5:1) and Congo red alone (Figure 9C). Incubation of T24 cells for 3 h with CR60-BAY caused a slight decrease in pErk1/2, which increased in those cells treated with this compound for 6 h. Similarly to RT4 cells, we did not observe a significant difference between treated and control T24 cells in total Akt and Erk1/2 (Figure 9B,D).

### 2.6. Analysis of Aggregates Formation

To uncover the effects of interaction between Congo red and sorafenib, probes with different molar ratios were prepared and measured by DLS, UV-VIS, and electrophoresis.

**Figure 10:**
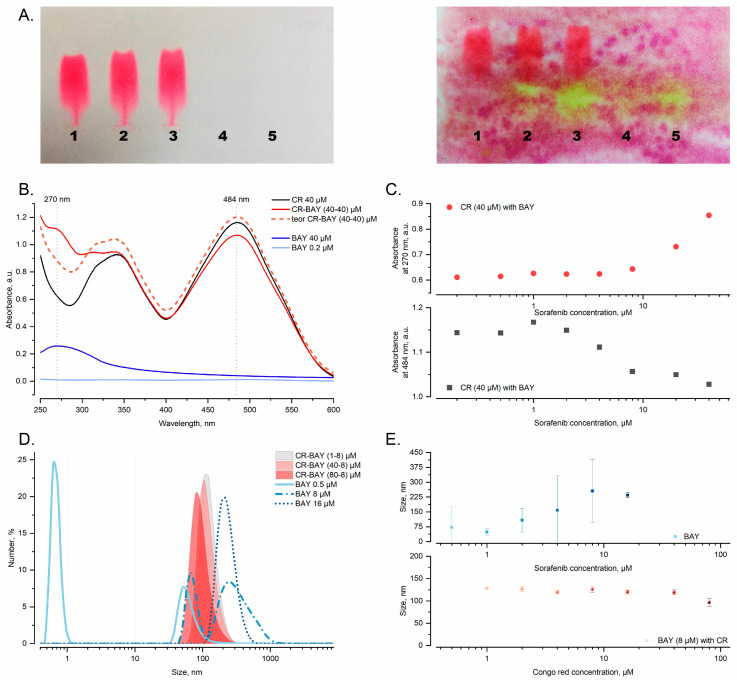
Analysis of aggregates formation. (**A**) Electrophoretic separation in 1% agar gel of samples is shown. The samples include (1) CR, (2) CR-BAY aggregates at a ratio of 5:1 and (3) 2-1, (4) BAY (as control) at a ratio of 5-1, and (5) 2-1. The CR concentration among all samples was kept constant at 40 µM. The gel is presented as a paper blot on the left panel and, after development, with potassium permanganate on the right panel. (**B**) UV-VIS spectra for selected CR-BAY aggregates ratios and varying BAY concentrations. The dashed line indicates the theoretical absorbance when BAY (40 µM) is added to CR (40 µM). (**C**) The absorbance at maxima wavelengths (270 nm in the upper panel and 484 nm in the bottom panel) is plotted for CR-BAY aggregates against changing concentrations of BAY. (**D**) DLS measurements, the size distribution by number prevalence for selected samples is depicted. The line profiles represent BAY at different concentrations, while the filled curves represent aggregates of CR and BAY at different ratios. (**E**) DLS measurements of BAY and CR-BAY series. The average size with standard deviation is plotted against the concentration of two substances, sorafenib (upper panel) and Congo red (CR), with a constant amount of sorafenib (8 µM, bottom panel).

To assess the presence of interactions between CR and BAY, we employed agarose gel electrophoresis as a simple method. Electrophoretic migration of CR was altered in the presence of aggregates with sorafenib. In particular, the CR-BAY aggregates migrated at a rate similar to free CR (Figure 10A, left panel). After applying a potassium permanganate solution, it became evident that the drug remained at the loading position, but the range of concentrations in (Figure 10A, right panel).

UV-vis measurements provide insights into the interactions primarily from the perspective of CR. The absorption spectrum of CR displays a strong absorption band in the range of 450–550 nm. Previous studies have demonstrated that a decrease in absorption within this range is indicative of nascent supramolecular complexes involving Congo red and ligands under examination [36]. The decrease in absorption at 484 nm observed for increased BAY-43-9006 concentration follows a characteristic sigmoidal form typical for intramolecular interaction. (Figure 10C, bottom panel).

Figure 10B demonstrates the decrease in absorption within the aforementioned range while also exhibiting an increase in absorption at 270 nm. The observed absorption at 270 nm is higher than the additive absorption of the drug and CR (dashed line). This increase in spectral properties indicates that the interaction between Congo red and the drug (BAY) induces changes that cannot be explained solely by the simple sum of the properties of the individual components. These alterations in the spectral characteristics suggest a molecular interaction between the two substances, leading to the formation of aggregates with unique features (Figure 10C, upper panel).

Although a large 15% change in the absorption value at 484 nm is an indisputable effect indicating the presence of interaction, it is impossible to determine the binding parameters on this basis due to a very aggregate process with unknown stoichiometry. This process appears to be multi-stage, as the observed change in absorption at 270 nm takes place at higher concentrations.

DLS analysis indicated that the sorafenib dilution series in PBS form aggregates with a polymodal size distribution with an average hydrodynamic diameter ranging from 50 nm (0.5–1 µM) to 500 nm (8–16 µM) (Figure 10E, upper panel). The presence of not-aggregated molecules was noticed only for the concentration of 0.5 µM (~7 Å), Figure 10D lined curves. The polydispersity index was measured to be 0.49 ± 0.18 on average for all measured probes of BAY 43-9006.

A series of aggregate dilutions were prepared by keeping the concentration of sorafenib constant while varying the concentration of Congo red. The filled curves in Figure 10D show the size distribution of the supramolecular aggregates of Congo red and sorafenib. The presence of Congo red can be observed to stabilize the resulting aggregates, as depicted in the distribution, as depicted in Figure 10E, lower panel. These stabilized aggregates exhibit a consistent size of approximately 125 nm, with a small standard deviation and weak dependence on CR concentration (Figure 10E, lower panel). 

## 3. Discussion

Given the poor prognosis of advanced bladder cancer and the limited effectiveness of current chemotherapeutics, intensive research continues on new pharmacological agents that may be promising in future targeted therapies for bladder cancer [37,38,39,40,41]. One such compound, sorafenib, is an oral multitarget inhibitor of various receptor tyrosine kinases involved in tumor progression [26]. Sorafenib has already been administered in vivo, in phase II clinical trials to patients with urothelial cancer [42,43], and in combination with cisplatin and gemcitabine in patients with bladder cancer [44]. Unfortunately, even this chemotherapeutic approach is unsatisfactory due to its high toxicity, again justifying the need for new targeted therapies [28,45,46].

Our in vitro studies demonstrate that sorafenib inhibits bladder cancer cell proliferation, induces apoptosis, and reduces cell migration and invasion. The results of our study are consistent with the results of other research groups that also found a decrease in the viability and survival of bladder cancer cells under the influence of sorafenib [27,28,47]. The observed effect was very pronounced, but the range of concentrations in which this drug works seems to be too high. The consequence of this is the side effects that we have demonstrated in normal uroepithelial SV-HUC-1 cells. Our studies in normal cells have shown that sorafenib inhibits proliferation and induces apoptosis at its therapeutic concentrations. Additionally, it is cytotoxic at higher concentrations.

The addition of Congo red to sorafenib (in the optimal molar ratio of 5:1) introduced by us led to a reduction in the concentration of the drug necessary to obtain the effect in vitro. It also led to a very clear selectivity of its action. Our studies have shown that the tested compound CR-BAY 5:1 reduces the cytotoxicity of sorafenib alone in normal urothelial cells and also protects them against apoptosis. Interestingly, CR-BAY 5:1 still inhibits the viability and survival of cancer cells, regardless of their malignancy. In addition, CR-BAY 5:1 also inhibits the migration and invasion of bladder cancer cells. It should be noted that Congo red alone had no significant effects on both bladder cancer cells and normal uroepithelial cells.

Our studies showed changes in the organization of the actin cytoskeleton and nanomechanical properties under the influence of sorafenib and its aggregate with Congo red. We showed changes in the value of Young’s modulus and observed the development of stress fibers at the bottom of tumor cells under the influence of sorafenib alone and its aggregate with Congo red. It should be noted that cancer cell elasticity is considered a unique mechanical marker of the cell’s metastatic abilities [48]. By far, the highest contribution to the elasticity of cells is from the cytoskeleton, in particular, the actin cytoskeleton [49,50,51]. Moreover, in cells with actin stress fibers, these structures significantly modify cells’ elasticity, as shown in the following work [52].

As the results of our research show, the joint administration of sorafenib and Congo red (in a molar ratio of 5:1) can be a very good therapeutic pathway. During our research, we constantly asked ourselves: why does CR (at a molar ratio of 5:1) make BAY more effective? Two explanations were possible: one—both compounds act independently on some pathways in the cell and intersect, leading to the observed synergy, or two—CR can stabilize BAY or create supramolecular structures that can better recognize cancer cells. The latter option, of course, does not exclude the first possibility.

Previous studies have shown that both compounds have the ability to penetrate cells [35,53] and cause changes in signaling pathways. Our study demonstrated that treatment of RT4 and T24 bladder cancer cells with sorafenib or CR-BAY 5:1 reduced Akt and Erk phosphorylation, which may be closely related to the decrease in proliferation and induction of apoptosis we observed. Previous studies have shown that sorafenib can also reduce mTOR and MAPK phosphorylation levels, as well as expression levels of MMP-2 and MMP-9 [42,54].

However, we still did not know whether these compounds act independently, triggering processes that act synergistically and, in the case of healthy cells, lead to cell survival in the presence of these compounds, or whether these compounds form supramolecular structures that somehow interact, for example, causing greater cell penetration cancerous than healthy. To verify this, studies on the interaction of these compounds were carried out, which confirmed the formation of structures outside the cell. 

Recent studies have underscored the expansive potential of nanoparticles and aggregates across diverse applications, particularly their promising role in tumor therapies [32,55,56,57,58,59].

The rationale behind employing Congo red for the stabilization of sorafenib demands elucidation to ensure clarity. Previous work has shown that azo-dyes like Congo red, which form aggregates, can stabilize colloids [31]. Aggregates of sorafenib were stabilized using the supramolecular dye Congo red, resulting in monodispersed colloids with medium diameters (50–100 nm). Aggregates with drug-to-dye ratios as high as 25:1 remained stable for 72 h in both buffers and serum-containing media. The addition of azo-dyes endowed these colloids with additional negative surface charges. These led to electrostatic repulsion, thus potentially driving the colloidal stability of these formulations [29].

Our initial research fills a gap in in vitro studies of these aggregates’ pharmacological potential. We confirmed that Congo red stabilizes sorafenib aggregates, improving homogeneity and reducing particle size. Congo red stabilization of BAY aggregates, potentially accounting for their effective action and selectivity according to in vitro studies. Our findings align closely with prior research [29,30,31,32,56]. Duan et al. demonstrated that sorafenib aggregates inhibit enzyme activities through surface adsorption. Co-aggregation with Congo red can mitigate this, allowing for precise activity tuning of different enzymes. Congo red—sorafenib aggregates also exhibit specificity towards absorbed proteins [29,30].

Another noteworthy insight arises from the study by the Lak et al. team, revealing a remarkable deceleration in the onset of sorafenib inhibition when co-aggregated with Congo red [30]. These findings establish a robust foundation for further exploration, aiming to elucidate the mechanisms underlying observed differences in the effects of these aggregates on cell lines in our studies.

Congo red emerges as a crucial factor influencing the performance of the resulting aggregate. Excessive aggregation of BAY may impede cell contact, hindering the drug’s efficacy. Conversely, optimal molar ratios between Congo red and the drug are pivotal, as research indicates that an excessively high concentration of Congo red can diminish the anti-cancer effects of the drug itself [31,32,60].

The described issue is extremely interesting and worth further research. The use of heterogeneous supramolecular systems is a path that cannot be missed.

## 4. Materials and Methods

### 4.1. Cell Culture

Human bladder cancer cell lines RT4 and T24 were obtained from the American Type Culture Collection (Manassas, VA, USA) and maintained in McCoy’s 5a medium (BioWest, Riverside, MO, USA) with fetal bovine serum (FBS, EURx Sp. z o.o., Gdansk, Poland) and 1% penicillin—streptomycin (BioWest, Riverside, MO, USA) at 37 °C in 5% CO_2_. An immortalized cell line from the urothelium of a human urinary bladder (SV-HUC-1) was obtained from the American Type Culture Collection (Manassas, VA, USA) and maintained in Ham’s F-12 medium (ATCC, Manassas, VA, USA) containing 10% FBS and 1% penicillin–streptomycin at 37 °C and 5% CO_2_. The cells were then passaged using 0.05% trypsin (BioWest, Riverside, MO, USA).

### 4.2. Treatments of the Cells

Sorafenib stock solutions (MedChem Express, Monmouth Junction, NJ, USA) were prepared by dissolving the compounds in dimethylsulfoxide (DMSO) (Sigma, St. Louis, MO, USA) at a final concentration of 10 mM. The final solutions were stored at −20 °C. McCoy’s 5a medium was used for the dilution of the stock solutions to obtain the working concentrations (sorafenib: 0.5–75 μM). As a negative control, served cells were cultured under analogous conditions at McCoy’s medium, whereas the positive control cells were cultured with taxol (PTX, taxol; Sigma, St. Louis, MO, USA) in a concentration of 50 nM. Congo red (5 mM) in PBS (pH 7.4) was heated to 100 °C for 2 min and then slowly cooled to room temperature for about 10 min after dilution using PBS. Sorafenib with diluted CR was prepared above to achieve the appropriate concentrations described in the text.

### 4.3. Cell Metabolic Viability Assays

All cells were seeded on 96-well plates at concentrations of 2 × 10^3^ cells/well. After 24-h growth at 37 °C in a humidified atmosphere, the culture medium was removed, and sorafenib (in concentration ranges 0.5–75 µM) or analyzed aggregates with Congo red (at concentration 60 µM or molarity ratio 5:1) were added to the cells. Incubation continued for the next 24, 48, and 72 h at 37 °C in a humidified atmosphere. The MTS assay (Promega, Madison, WI, USA) [61] was used to determine the compounds influence of the tested on the viability of bladder cancer cells (RT4, T24) and human uroepithelial SV-HUC-1 (normal) cells. The absorbance was measured at 570 nm using an Epoch Microplate Spectrophotometer (BioTek Instruments Inc., Winooski, VT, USA). The effect of sorafenib or CR-BAY aggregates was presented as the proliferation curves [62].

The equation for the Gi (growth inhibition) parameter was taken from the paper by Bojko et al. [63].

Gi = (Ai − A0)/(Ak − A0) × 100%

where:

- Gi is the growth inhibition,
- Ai is the average absorbance after incubation with the compounds,
- A0 is the average absorbance of the control at the start of the experiment,
- Ak is the average absorbance of the control after incubation without the compounds.

### 4.4. Cell Survival Analysis by Flow Cytometry

Apoptosis was analyzed with PE Annexin V Apoptosis Detection Kit I (BD Biosciences, San Jose, CA, USA), following the manufacturer’s instructions. Briefly, RT4, T24, or SV-HUC-1 cells (3 × 10^5^) were seeded and cultured overnight on a six-well plate with investigated compounds for 48 h and 72 h. Approximately 1 × 10^5^ cells were stained with Annexin V for 15 min at RT in the dark. After the incubation, 5 μL of propidium iodide and 400 μL of buffer solution were added prior to the cytometric analysis alone. Fluorescence intensities of treated samples and controls were analyzed by flow cytometry on a BD FACS CantoTM instrument (Becton Dickinson, New York, NY, USA). Experiments were performed at least twice.

### 4.5. Caspase-3 Activity Assay

Apoptosis evaluation also included measuring caspase 3 activity. Briefly, cells were placed and grown in 24-well plates, then treated with compounds for 24 and 48 h. Subsequently, cells were lysed with a 0.05 M Tris/HCl buffer (pH 7.6) containing 0.1 M NaCl, 0.001 M EDTA, and 1% Triton X-100 for caspase-3 activity (DEVD-like caspase activity). Concurrently, a similar experiment was conducted to determine cell count in each well using MTS. The release of aminofluorocoumarin (AFC) during the reaction with Ac-DEVD-AFC was quantified with a spectrofluorometer. For ease of interpretation, the fluorescence indicative of caspase-3 activity was standardized to the cell count (DEVD-like caspase activity per MTS cell number) and presented as a multiple of the increase compared to the control group. The results show a single experiment performed in triplicate.

### 4.6. Cell Cytotoxicity by LDH Assay

After 24h of SV-HUC-1 cell line, the medium supernatant was collected in order to evaluate the cytotoxicity of the investigated compounds by the lactose dehydrogenase (LDH, Thermo Fisher Scientific, Waltham, MA, USA) assay. Into wells with 50 μL of each sample medium, aliquots 50 μL of LDH assay buffer were added, and the plates were transferred to the incubator for 30 min. The reaction was terminated by adding 50 μL of stop buffer. The absorptions were read at wavelengths of 490 nm and 680 nm using a Synergy_HT plate reader (Biotek Instruments, Winooski, VT, USA). Cell death was inferred by subtracting the 680 nm values from the 490 nm ones.

### 4.7. Scratch Assay

Confluent RT4 and T24 cells were cultured with McCoy medium with 0.5% BSA for 24 h. Next, cells were treated with investigated compounds for 24 h. The control cells were cultured under identical conditions without BAY-43-9006 and CR. The monolayer was scratched with a pipette tip and washed with phosphate-buffered saline (PBS) to remove floating cells. The scrape was monitored and photographed after 24 h incubation. The pictures were analyzed using FiJi Image J 1.54f software (National Institute of Health, Bethesda, MD, USA), as described previously [64].

### 4.8. Chemotaxis and Invasion through Matrigel

Chemotaxis of bladder cells to 10% FBS in MCoy’s medium was evaluated using a modified Boyden’s chamber with 8 μm pore polycarbonate membrane inserts (Transwell; Corning Life Sciences, PZ HTL SA, Warsaw, Poland), and the chemotactic cells were visualized with Wright’s staining (Sigma-Aldrich, Darmstadt, Germany), as previously described [64]. BSA MCoy’s medium 0.5% was used as a negative control. Before the experiment, RT4 and T24 cells were treated with sorafenib at IC50 concentration or CR-BAY aggregates for 24 h. 2.0 × 10^4^ RT4 and T24 cells in 0.5 mL in starving medium 0.5% BSA MCoy’s with or without the inhibitor were seeded per one insert. Bladder cancer cells’ invasion ability was measured in Matrigel-coated Transwell insert chambers (Corning LifeSciences, PZ HTL SA, Warsaw, Poland). The inserts were coated with 1 mg/mL Matrigel matrix according to the manufacturer’s recommendations. Methods used in the cell invasion assay were similar to those in the cell chemotactic assay.

### 4.9. Cell Elasticity Measurements

Nanomechanical analysis of cells was conducted using a Bioscope Catalyst (Bruker) atomic force microscope (AFM) coupled with an inverted optical microscope Axio Observer Z1 from Zeiss. During the analysis, cells were maintained in a culture medium and kept under 37 °C. Elasticity measurements of cells were made employing force spectroscopy mode that consists of measuring force-displacement curves [48]. In the analysis, a soft cantilever with a nominal tip radius of 20 nm and with an experimentally determined spring constant of 0.012 N/m (Bruker Probes was used). Before measuring a cell, the AFM probe was positioned on top of a cell and aligned at the cell center using an optical microscopy view. Once aligned, force-displacement curves from a grid of 5 × 5 points were collected at a rate of 1 Hz. A total of 20 cells for each experimental group were analyzed. A detailed description of the nanomechanical analysis used in this work can be found elsewhere [48]. The obtained force-displacement curves were analyzed using the AtomicJ_2.3.1software [65]. 

### 4.10. Cytoskeleton Analysis

Bladder cancer cells were cultured on glass coverslips for 48 h in the presence of sorafenib and CR-BAY 5:1. After this time, cells were rinsed three times with Dulbecco’s Phosphate Buffered Saline (DPBS, Sigma-Aldrich, Darmstadt, Germany) and fixed with 4% paraformaldehyde for 20 min. Permeabilization of the cell membrane was achieved by the treatment of fixed cells with cool (4 °C) 0.2% Triton X-100 for 5 min. Actin filaments were stained with phalloidin conjugated with Alexa Fluor 488 dye (0.33 μM; A12379, Invitrogen, Eugene, OR, USA) for 1hr at RT in the dark. After washing with DPBS three times, 0.2 µg/mL Hoechst 33342 (H3570; Invitrogen) for staining the nucleus was added and incubated for 15 min at RT in the dark. Analysis of the cell cytoskeleton was made using scanning laser confocal microscopy (Zeiss LSM 900 with Airyscan 2), as described in the following work [51]. In brief, the lowest and highest position of a cell was determined, and a ‘z-stack’ was obtained. A total of 10 cells for each experimental group were analyzed.

### 4.11. Western Blot Analysis

Bladder cancer cell lines were seeded on 6 cm plates in the 80% confluence and were serum-deprived for 12 h before stimulation. Cells were exposed to sorafenib (IC50 concentration) and analyzed aggregates with Congo red for 3 or 6 h. The control plates were non-treated cells in a standard culture medium.

Cells were washed with cold phosphate-buffered saline solution (PBS, Sigma-Aldrich Corp., St. Louis, MO, USA) and lyzed in buffer containing 0.0625 M Tris/HCl pH 6.8, 2% SDS, 10% glycerol, 5% β-mercaptoethanol (Sigma-Aldrich Corp., St. Louis, MO, USA). Cells were dislodged from the culture dish using a scraper. The mixtures were pipetted into Eppendorf’s tubes and kept on ice. Samples were then boiled for 5 min at 95 °C and sonicated 3 × 5 s (Bandelin Sonoplus GM 70, Bandelin electronic GmbH and Co. KG, Berlin, Germany). Samples prepared in this way were stored at −80 °C until analysis. A Qubit^®^ Protein Assay Kit (Thermo Fisher Scientific, Pierce Biotechnology, Rockford, IL, USA) was used to determine the protein concentration of samples. Cell lysates containing equal amounts of protein (20 µg) were separated on 10% SDS-PAGE gels and subsequently transferred onto 0.22 µm PVDF membranes (Avantor Performance Materials Poland S.A., Gliwice, Poland). After blocking in 5% non-fat milk in Tris-Buffered Saline Tween-20 (TBST, Sigma-Aldrich Corp., St. Louis, MO, USA) for 1 h, membranes were incubated in the primary antibodies overnight (Table 2).

Following, the membranes were incubated with horseradish peroxidase (HRP)-conjugated goat anti-rabbit (#7074) or horse anti-mouse (#7076) IgG secondary antibody (1:3000) (Cell Signaling Technology Inc.). Protein immunoreactivity was detected by chemiluminescence, and images were recorded with a ChemiDocTM MP Imaging System (Bio-Rad, Hercules, CA, USA). Molecular masses were estimated by reference to standard proteins (Thermo Fisher Scientific, Pierce Biotechnology, Rockford, IL, USA). Hsp90 served as a loading control. Densitometric analysis of proteins was performed using an Image Lab Software, version 6.0 (Bio-Rad, Hercules, CA, USA). Representative membranes from at least three independent experiments with similar results are shown.

### 4.12. Sample Preparation

Congo red (5 mM; TCI America, Portland, OR, USA) in PBS (pH 7.4; Eurx, Gdansk, Poland) was heated to 100 °C for 2 min and then slowly cooled to room temperature for about 10 min after dilution using PBS. Sorafenib (BAY-43-9006, BAY; MedChem Express, Monmouth Junction, NJ, USA) solved in DMSO (10Mm; Sigma, St. Louis, MO, USA) was prediluted with pure water up to 1 mM and then mixed with CR diluted prepared above to achieve the appropriate concentrations described in the text.

### 4.13. Dynamic Light Scattering (DLS)

Dynamic light scattering (DLS) measurements were performed at 25 °C using a Zetasizer Nano ZSP equipped with a red (633 nm) laser (Malvern Instruments Ltd., Worcestershire, UK). A quartz cuvette (ZEN2112) QS 3.00 mm from Malvern Instrument was used for the measurements. Data were analyzed using Malvern Zetasizer 7.13 software (Malvern Instruments Ltd., Worcestershire, UK). All sizes reported here were based on the PSD number. For each sample, three DLS measurements were conducted with a fixed six runs, and each run lasted 10 s. A detection angle of 173° was chosen for the size measurement.

### 4.14. UV-VIS

UV-vis spectra were obtained on a carry 300 spectrophotometer (CarryWinUV, Perlan, Agilent Technologies, Inc., Santa Clara, CA, USA). The mode of measurement was scans ranging from 250 to 600 nm (200 nm/min, slit 3 nm).

### 4.15. Electrophoresis

Separation of the aggregates (CR-BAY) using electrophoretic forces was performed in a thin layer of 2% agarose gel. The electrode buffer was TRIS-HCl (pH 7.8). Separation was carried out under 180 V for 60 min. In addressing the lack of color and fluorescence, was employed a 0.5% potassium permanganate solution was employed to develop sorafenib spots on the blotting paper. Sorafenib oxidation products exhibited a distinctive yellow hue, enhancing visibility.

### 4.16. Statistical Analysis

Unless stated otherwise, the results show the mean ± standard deviation (SD) of at least three independent experiments. Statistical analysis was performed by one-way ANOVA with Dunnett’s post-test, the Mann–Whitney test, or Student’s *t* test using GraphPad Prism 5.01 software. Differences with a value of *p* < 0.05 were considered statistically significant. IC50 and IC_90_ values were obtained by fitting a sigmoidal model to the data showing the inhibitor curves for BAY-43-9006 using Origin 9.1.

## 5. Conclusions

This study contributes to understanding the interaction between CR and BAY and how this interaction can alter the observable effect on cells. We suppose that one of these features may be the stability of the complexes. Our studies have shown that CR-BAY (5:1) can reduce the cytotoxicity of sorafenib on healthy cells and protect them from apoptosis. Interestingly, it still has a strong anti-cancer effect on cancer cells. These findings may have implications for the development of new drug delivery systems or therapeutic approaches using these substances. However, further research is required to clarify the mechanisms of action of supramolecular CR-BAY structures with various types of cells.

## Figures and Tables

**Table 1:** Values of IC50 and IC90 obtained by fitting a sigmoidal model to the data showing the inhibitor curves for BAY-43-9006. Data are expressed as a mean ± standard error.

Cell Line	RT4		T24	
	IC50	IC90	IC50	IC90
BAY [µM]	8.01 ± 0.05	68.8 ± 0.07	8.45 ± 0.05	36.15 ± 0.17
CR-BAY (5:1) [µM]	10.04 ± 0.04	62.70 ± 0.12	6.18 ± 0.05	28.00 ± 0.12
CR60-BAY [µM]	40.22 ± 0.06	-	9.56 ± 0.06	40.46 ± 0.15

**Table 2:** List of the antibodies and conditions that were used in this study.

Primary Antibody	Material Number	Host Species	Dilution	Vendor
Akt	#9272	rabbit	1:1000	Cell Signaling Technology Inc. (Danvers, MA, USA)
phospho-AKT (Ser473)	#4060	rabbit	1:1000	
p44/42 MAPK (ERK1/2)	#4695	rabbit	1:1000	
phospho-p44/42 MAPK (Thr202/Tyr204) (phospho-ERK1/2)	#9106	mouse	1:1000	
Hsp90	610418	mouse	1:4000	BD Transduction Laboratories

## Data Availability

The datasets used and/or analyzed during the current study are available from the corresponding author upon reasonable request.

## Supplementary Materials

The following supporting information can be downloaded at: https://www.mdpi.com/article/10.3390/ijms25010269/s1.
