# Supplementary material for: The Potential of Congo Red Supplied Aggregates of Multitargeted Tyrosine Kinase Inhibitor (Sorafenib, BAY-43-9006) in Enhancing Therapeutic Impact on Bladder Cancer
Source: Int J Mol Sci. 2023 Dec 23;25(1):269. doi: 10.3390/ijms25010269 (PMC10779242; doi:10.3390/ijms25010269)
Supplement: Supplementary file 1 [file ijms-25-00269-s001.zip › ijms-2732291-supplementary.pdf]

The potential of Congo red supplied aggregates of multitargeted tyrosine kinase inhibitor (sorafenib, BAY-43-9006) in enhancing therapeutic impact on bladder cancer

Małgorzata Lasota, Daniel Jankowski, Anna Wiśniewska, Michał Sarna, Marta Kaczor-Kamińska, Anna Misterka, Mateusz Szczepaniak, Joanna Dulińska-Litewka, Andrzej Górecki

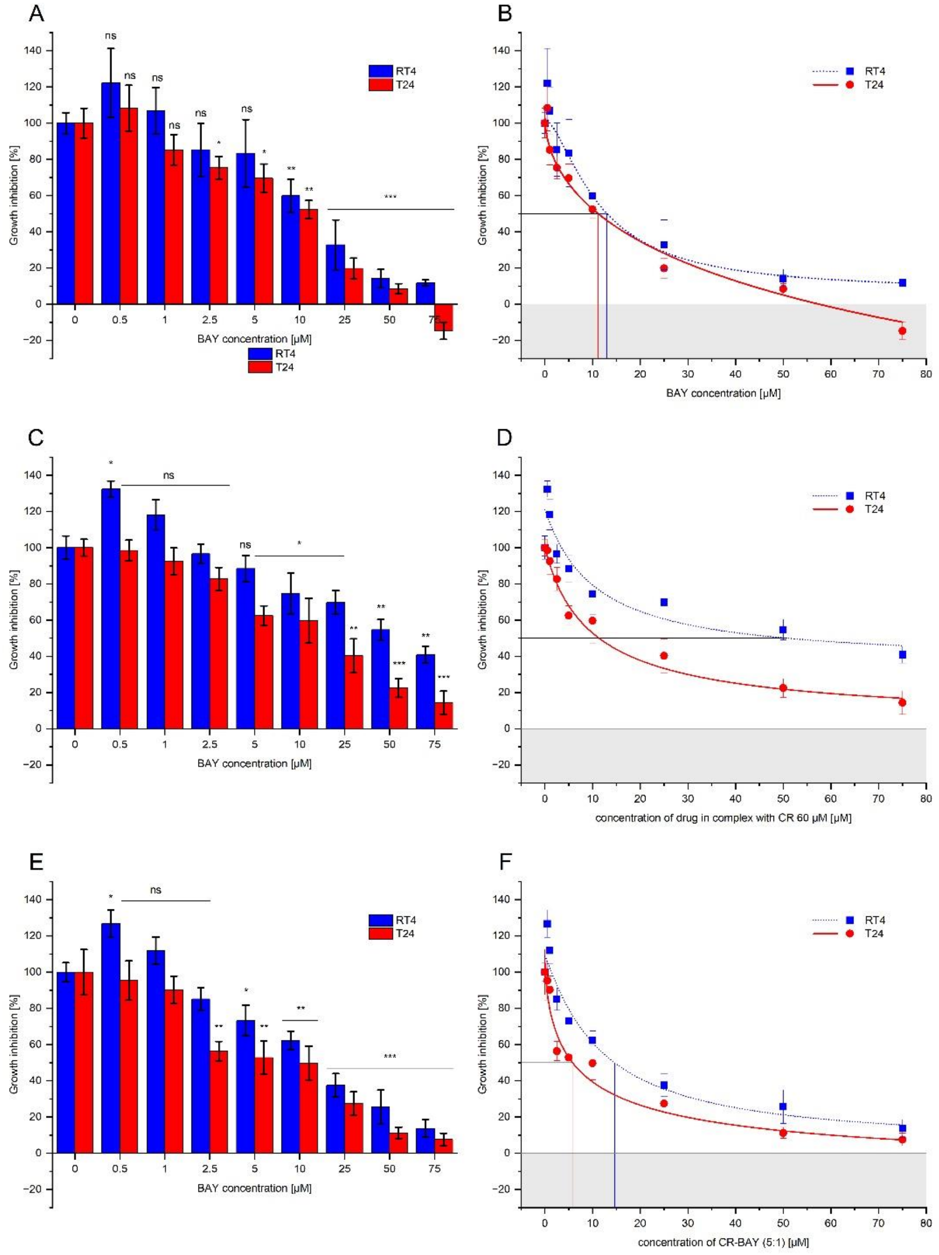

Supplementary Figure S1. Effect of sorafenib alone and in aggregates with Congo red on bladder cells (RT4 and T24). The dose-dependent effect of sorafenib alone and in aggregates with Congo red on the viability of RH30 and

RD cells after 24 h incubation (A, C, E). Statistical significance between non-treated and treated samples was evaluated using ANNOVA with Dunnett post-test: ns- non-significant (p > 0.05) in comparison with a control sample (without investigated compounds); *0.01 < p < 0.05, **0.001 <p < 0.01. Growth inhibition curve in standard culture conditions (B, D, F).

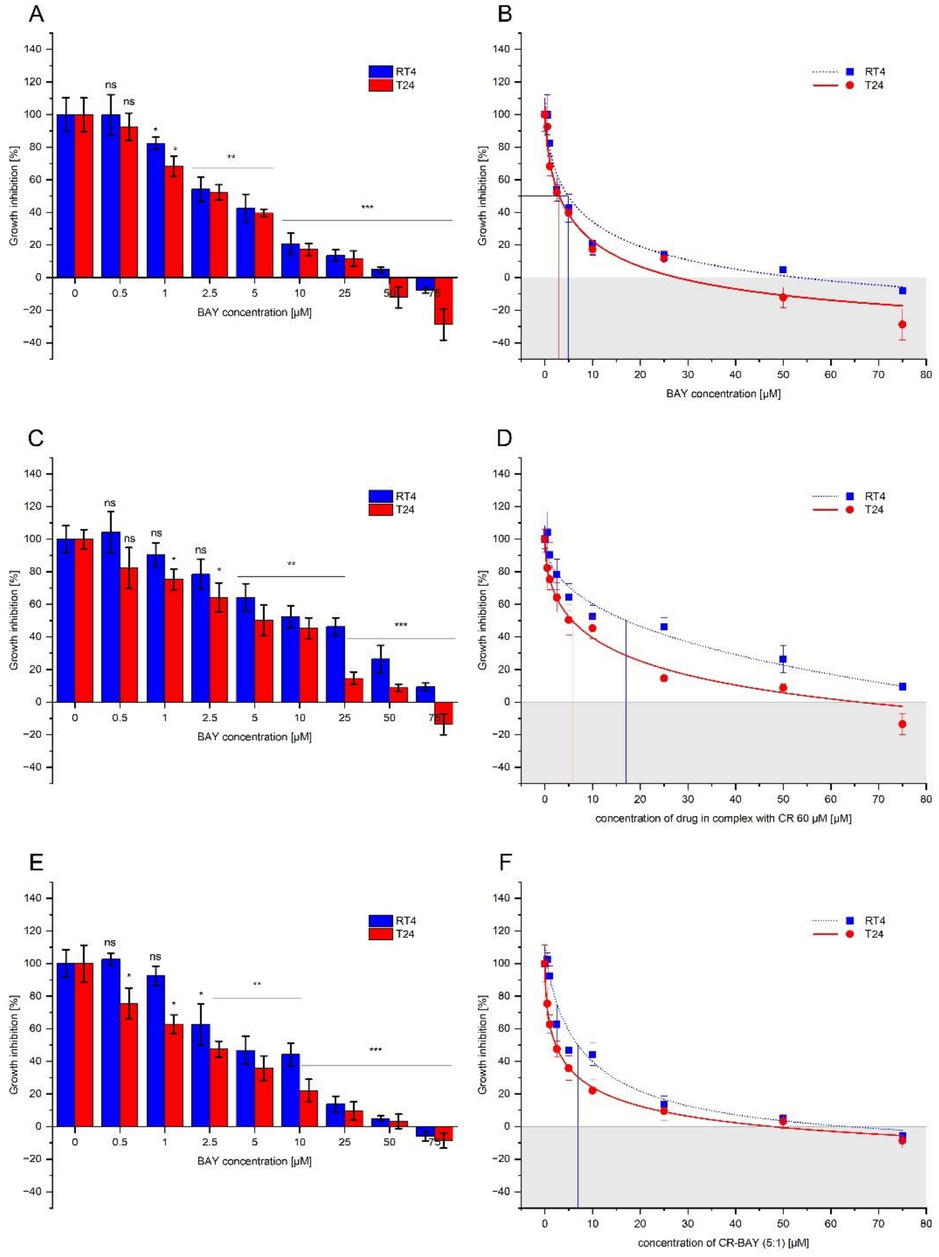

Supplementary Figure S2. Effect of sorafenib alone and in aggregates with Congo red on bladder cells (RT4 and T24). The dose-dependent effect of sorafenib alone and in aggregates with Congo red on the viability of RH30 and RD cells after 72 h incubation (A, C, E). Statistical significance between non-treated and treated samples was evaluated using ANNOVA with Dunnett post-test: ns- non-significant (p > 0.05) in comparison with a control sample (without investigated compounds); *0.01 < p < 0.05, **0.001 <p < 0.01. Growth inhibition curve in standard culture conditions (B, D, F).

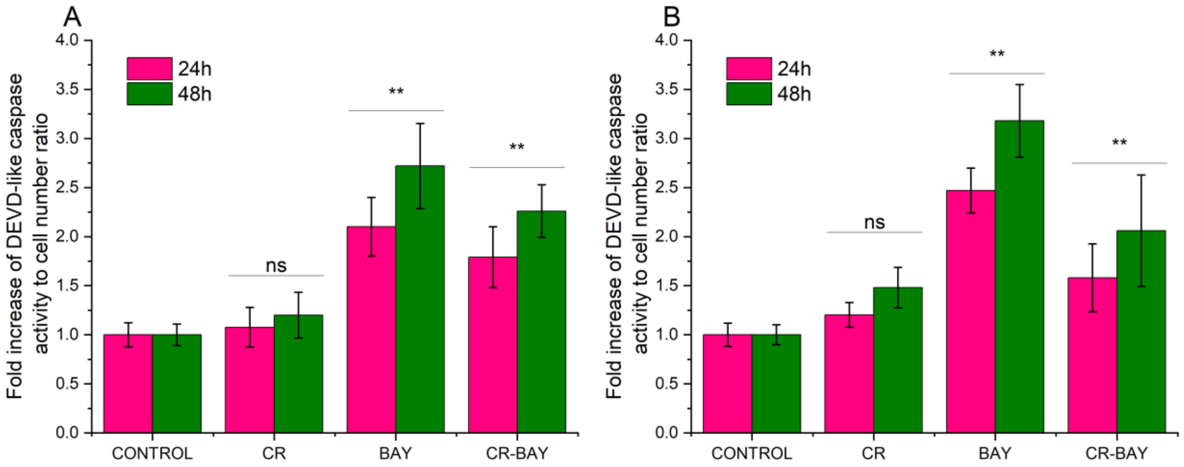

Supplementary Figure S3. Caspase-3 activation after 24 and 48 h in RT4 (A) and T24 (B) cells after stimulation with investigated compounds. The results were subjected to statistical analysis with Student's t-test: ns- non-significant ($p > 0.05$)
